# Ribociclib-induced Vitiligo: a Case Report

**DOI:** 10.5826/dpc.1202a45

**Published:** 2022-04-01

**Authors:** Nicolás Silvestre Torner, Antonio Aguilar Martínez, María José Echarri González, Sergio Tabbara Carrascosa, Jorge Román Sainz, Fernando Gruber Velasco

**Affiliations:** 1 Department of Dermatology, Hospital Universitario Severo Ochoa, Leganés (Madrid), Spain; 2 Department of Oncology, Hospital Universitario Severo Ochoa, Leganés (Madrid), Spain

**Keywords:** vitiligo, ribociclib, selective cyclin-dependent kinase 4/6 inhibitors, breast cancer, adverse event

## Introduction

Selective cyclin-dependent kinase 4/6 inhibitors (CDK 4/6i) – ribociclib, palbociclib and abemaciclib – are a novel therapeutic option for breast cancer [1]. CDK 4/6 is are well tolerated and have shown a manageable safety profile, with mild hematological, gastrointestinal and cutaneous adverse events (AE). Vitiligo-like lesions are a dermatologic AE exceptionally reported with CDK 4/6is use [2].

## Case Presentation

**Figure 1:**
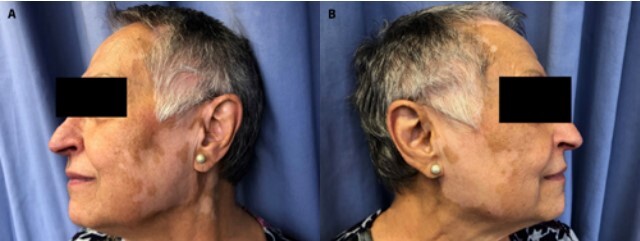
(A, B) Clinical images. Achromic sharply demarcated macules in the neck, cheeks and ears, with affectation of the hair follicle.

**Figure 2:**
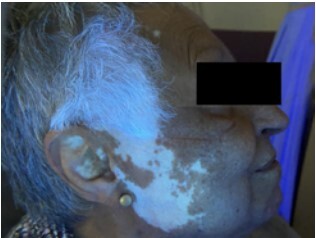
Clinical image. Bright white and sharply delineated lesions showed with Wood lamp.

A 70-year-old woman presented with a 6-week history of asymptomatic facial hypopigmented spots. For the last 8 months, she was receiving treatment with letrozole and ribociclib for a hormone receptor-positive (HR-positive) and human epidermal growth factor receptor-2-negative (HER2-negative) metastatic breast cancer. Dermatological examination revealed unpigmented, well-defined macules distributed symmetrically in the neck and the face, with affectation of hair follicle (Figure 1, A and B). The Wood lamp examination showed bright white and sharply delineated lesions (Figure 2). Based on these findings, a diagnosis of vitiligo was made.

## Discussion

Vitiligo is an acquired pigmentary autoimmune disorder consisting of the development of hypopigmented macules due to the selective loss of melanocytes. The cause of vitiligo remains unknown. Vitiligo-like lesions have been reported as an AE in oncological patients treated with anti-programmed death-1/programmed death-ligand 1 (PD-1/PD-L1) immunotherapies (pembrolizumab, nivolumab), as well as in patients treated with tyrosine-kinase inhibitors (imatinib, cabozantinib, pazopanib) [2]. It is considered an indicator of survival benefit in melanoma patients treated with anti-PD-1/PD-L1 immunotherapies.

Selective CDK 4/6is are currently approved by the US Food and Drug Administration and the European Medicines Agency for the treatment of patients with HR-positive, HER2-negative advanced or metastatic breast cancer. Hematologic toxicity, gastrointestinal disturbances and fatigue are the most frequent side effects of this class of agents [1]. The most common dermatological adverse event is alopecia, which might be increased by the association of endocrine therapy. Moreover, pruritus and a maculopapular rash have also been reported as cutaneous adverse reactions in patients treated with ribociclib [1,2].

Vitiligo-like lesions have been described in patients treated with CDK 4/6is too, mostly in relation to ribociclib [2]. Although the pathogenic mechanism between CDK 4/6is and vitiligo is still unclear, it has been classified as a class-related AE. The cell-cycle arrest and consequent apoptosis induced by CDK 4/6is [1], may lead to a premature death of melanocytes, that clinically manifests as achromic lesions. The prognostic meaning of vitiligo lesions in patients treated with CDK 4/6is remains still unclear.

Treatment of vitiligo induced by CDK 4/6i is challenging. Similar therapeutic strategies followed in other vitiligo patients can be performed. However, immunosuppressants and biological therapies should be avoided in oncological patients. Partial response has been achieved with topical immunosuppressants in combination with oral corticosteroids [2].

## Conlusions

Depigmentation may cause psychological distress and may decreased quality of life. Therefore, oncological patients treated with CDK 4/6is should be informed about this potential AE and should be referred to a dermatologist for accurate diagnosis and treatment.
